# Rapid De Novo Evolution of X Chromosome Dosage Compensation in *Silene latifolia*, a Plant with Young Sex Chromosomes

**DOI:** 10.1371/journal.pbio.1001308

**Published:** 2012-04-17

**Authors:** Aline Muyle, Niklaus Zemp, Clothilde Deschamps, Sylvain Mousset, Alex Widmer, Gabriel A. B. Marais

**Affiliations:** 1 Laboratoire de Biométrie et Biologie Evolutive (UMR 5558), CNRS/Université Lyon 1, Villeurbanne, France; 2 Institute of Integrative Biology (IBZ), ETH Zurich, Zürich, Switzerland; 3 Pôle Rhône-Alpes de Bioinformatique (PRABI), Villeurbanne, France; Max Planck Institute for Developmental Biology, Germany

## Abstract

*Silene latifolia* is a dioecious plant with heteromorphic sex chromosomes that have originated only ∼10 million years ago and is a promising model organism to study sex chromosome evolution in plants. Previous work suggests that *S. latifolia* XY chromosomes have gradually stopped recombining and the Y chromosome is undergoing degeneration as in animal sex chromosomes. However, this work has been limited by the paucity of sex-linked genes available. Here, we used 35 Gb of RNA-seq data from multiple males (XY) and females (XX) of an *S. latifolia* inbred line to detect sex-linked SNPs and identified more than 1,700 sex-linked contigs (with X-linked and Y-linked alleles). Analyses using known sex-linked and autosomal genes, together with simulations indicate that these newly identified sex-linked contigs are reliable. Using read numbers, we then estimated expression levels of X-linked and Y-linked alleles in males and found an overall trend of reduced expression of Y-linked alleles, consistent with a widespread ongoing degeneration of the *S. latifolia* Y chromosome. By comparing expression intensities of X-linked alleles in males and females, we found that X-linked allele expression increases as Y-linked allele expression decreases in males, which makes expression of sex-linked contigs similar in both sexes. This phenomenon is known as dosage compensation and has so far only been observed in evolutionary old animal sex chromosome systems. Our results suggest that dosage compensation has evolved in plants and that it can quickly evolve de novo after the origin of sex chromosomes.

## Abstract 2

Evidence for dosage compensation in *Silene latifolia*, a plant with 10-million-year-old sex chromosomes, reveals that dosage compensation can evolve rapidly in young XY systems and is not an animal-specific phenomenon.

## Author Summary

The mammalian sex chromosomes originated from an ancestral pair of autosomes about 150 million years ago and the Y chromosome subsequently degenerated, losing most of its genes. During this process, a phenomenon called dosage compensation evolved to compensate for the gene loss on the Y chromosome and to equalize expression of X-linked genes in the two sexes. In humans, this is achieved by inactivating one of the two X chromosomes in females. Dosage compensation has also been reported in other animal XY systems such as fruit flies and worms, each 100 million years old or more. Here we studied dosage compensation in plants. We used high-throughput RNA sequencing in male and female *Silene latifolia* (white campion)—a dioecious plant whose XY chromosomes originated only about 10 million years ago—to identify hundreds of sex-linked genes. Analysis of their expression patterns in males and females revealed equal doses of sex-linked transcripts in both sexes, regardless of the degree of reduction of Y expression due to degeneration. Our results thus show that dosage compensation occurs in plants and is thus not an animal-specific phenomenon. They also reveal that proportionate dosage compensation can evolve rapidly de novo after the origin of sex chromosomes.

## Introduction

In humans, where the evolution of sex chromosomes is probably best known, the XY chromosome pair was originally a recombining pair of autosomes that progressively stopped recombining, most likely because of a series of inversions on the Y chromosome [1]–[4]. This started ∼150 million years ago [5],[6] and the non-recombining human Y chromosome subsequently suffered from degenerating processes known as Hill-Robertson effects (inefficient selection and reduced polymorphism, see [7]–[9]), which explain the massive loss of Y genes (∼97%) and the concomitant accumulation of DNA repeats on the non-recombining Y compared to the X chromosome and the still recombining pseudoautosomal regions (PARs) [2],[3]. Even the few genes that persisted on the Y show signs of degeneration [10],[11]. The classical view is that the massive loss of Y-linked genes has been balanced by the evolution of dosage compensation (equal dosage of X and autosomal transcripts in both males and females [12]–[14]), which is achieved by the inactivation of one X chromosome in females [15]. The question whether this three-step scenario (X–Y recombination suppression, Y degeneration, X dosage compensation) is similar for all species with sex chromosomes, in particular those with much younger sex chromosomes, has received much attention from evolutionary biologists, and several alternative model organisms to study the evolution of sex chromosomes have emerged, some of them very recently [9],[16]–[18].

*S. latifolia* (white campion) is one such model organism. It is a dioecious plant from the Caryophyllaceae family with heteromorphic sex chromosomes that have originated only ∼10 million years ago [19]–[22] and is a promising model organism to study sex chromosome evolution in plants [23],[24]. Previous work suggests that *S. latifolia* XY chromosomes have stopped recombining gradually [21],[22],[25] and that the Y is undergoing degeneration (gene loss, reduced polymorphism, accumulation of repeats, maladapted proteins, reduced gene expression) as in animal sex chromosomes [26]–[34]. Despite these highly interesting results, work on sex chromosome evolution in *S. latifolia* has been limited by the slow pace of sex-linked gene identification (one to two new genes/year) [21],[25],[35]–[40]. This situation is now changing rapidly, thanks to next-generation sequencing (NGS) approaches, which are helping reveal the strong potential of the *S. latifolia* model [23],[24],[41]–[43].

Here we report a study using such an NGS approach, RNA-seq, applied to several males and females of an *S. latifolia* inbred line. Using a de novo assembly strategy followed by SNP analysis, we identified >1,700 sex-linked contigs, increasing by almost 100-fold the number of sex-linked sequences available until recently in *S. latifolia*. Studying these 1,700 sex-linked contigs, we found that expression of alleles on the Y is significantly reduced compared to those on the X chromosome, providing evidence for large-scale ongoing degeneration of the *S. latifolia* Y chromosome. By comparing the expression of X-linked alleles in males and females, which differ in the number of X chromosomes, we further found evidence of equal dosage of X transcripts among sexes for sex-linked genes showing Y degeneration, a phenomenon known as dosage compensation. To our knowledge, this is the first evidence for dosage compensation in plants and reveals that dosage compensation is not an animal-specific phenomenon. Moreover, the finding of dosage compensation in evolutionary young sex chromosomes has novel implications for the evolution of sex chromosomes because it shows that 10 million years are sufficient to evolve dosage compensation de novo. By contrast, dosage compensation in animals has to date been documented only in >100-million-year-old sex chromosome systems.

## Results

### Identification and Validation of New Sex-Linked Genes

We used RNA-seq—a next-generation transcriptome-sequencing approach—to identify new sex-linked genes and to study gene expression (find more details in Text S1). We obtained ∼35 Gb of sequence data from three males and three females from a ten-generation inbred population of *S. latifolia* using Illumina technology (Table S1). Male and female reads were pooled and assembled de novo (see Material and Methods) (Figure S1), and we obtained 141,855 contigs (Table S2). From these, we identified sex-linked contigs using a segregation analysis similarly to [42],[43] and found 1,736 contigs with at least one sex-linked SNP (Table S2). We tested the reliability of our inference of sex-linkage by first using known autosomal genes [44] to see whether sex-linked SNPs have been wrongly inferred for these, but could not find any for the ten autosomal genes tested (Table S3). This very low rate of false positives was confirmed when running our scripts to detect sex-linked SNPs on a set of simulated autosomal SNPs (Text S2). We thus concluded that our inferences of sex-linkage are highly reliable. To estimate how many sex-linked contigs we missed with our method, we checked how many of the previously identified sex-linked genes were among our sex-linked contigs (Table S3). 42% of these were not found, which means that our rate of false negatives is quite high, and we identified a subset (probably about half; see Figure S2; Text S1) of the sex-linked genes in *S. latifolia*. Many of our sex-linked contigs should be full-length transcripts as suggested by the size distribution plot (Figure S3).

### Expression Analysis of X-Linked and Y-Linked Alleles

**Figure 1:**
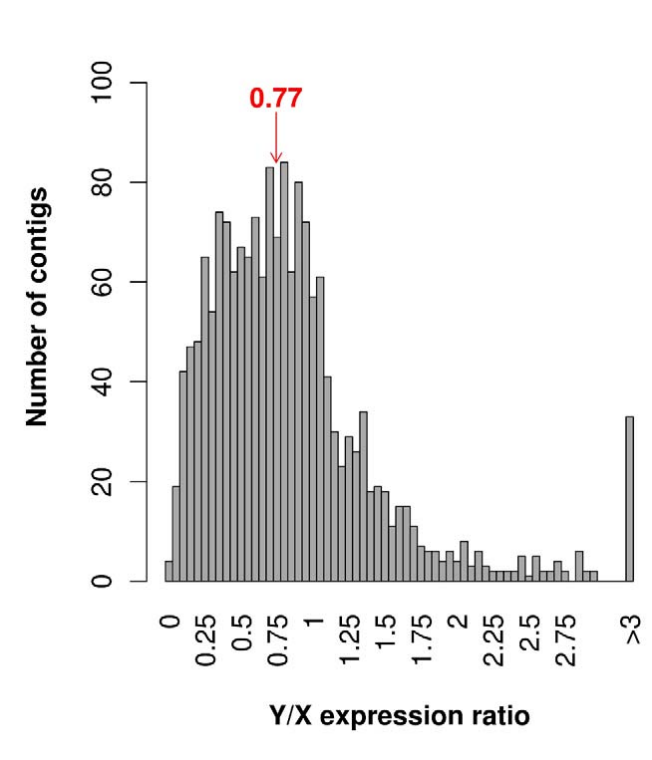
Distribution of Y/X expression ratios in *S. latifolia* males for the 1,736 sex-linked contigs. Total Y and X read numbers were summed at sex-linked SNP locations for each contig and normalized for each male separately, then averaged across males to obtain the Y/X ratio. The median is shown in red.

We used read numbers to estimate expression levels of the sex-linked contigs (see Material and Methods). We first compared expression levels of X-linked and Y-linked alleles in males. The read numbers were normalized to be able to combine data from different male individuals. As shown in Figure 1, we found that the Y/X expression ratio is significantly less than 1 (median 0.77, mean 0.89, significant Wilcoxon paired test *p*<10^−16^). This is in agreement with previous work on six experimentally identified sex-linked genes [33] and also with recent work using RNA-seq data [42],[43]. Why Y expression is reduced over evolutionary time is not fully understood. It could be because of the accumulation of slightly deleterious mutations in promoters and cis-regulatory elements, and/or the insertion of transposable elements when the methylation of these elements spreads to nearby genes. However, this trend is considered a hallmark of Y chromosome degeneration and has been observed in several animal systems [45],[46]. Y degeneration is thus clearly visible in *S. latifolia* but may not be as pronounced as expected because of haploid selection on pollen preventing the degeneration of many pollen-expressed Y genes [42] (but see [43],[47]).

**Figure 2:**
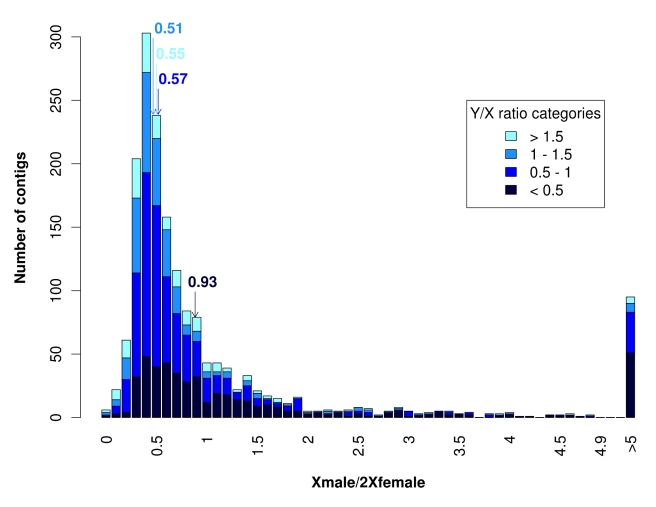
Distribution of the ratio between the expression of the single X in males and the two X copies in females (Xmale/2Xfemale) for all sex-linked contigs. Different categories of sex-linked contigs are shown: Y/X ratio below 0.5 (379 contigs), Y/X ratio between 0.5 and 1 (656 contigs), Y/X ratio between 1 and 1.5 (315 contigs), Y/X ratio above 1.5 (195 contigs). Medians are indicated in the colour corresponding to each Y/X ratio category. When the contigs with high Xmale/2Xfemale ratios are removed as in Figure 3 (see text for explanations) the medians remain unaltered except for the category Y/X<0.5 where it changes to 0.76 but is still significantly different from 0.5 (Wilcoxon test, *p*<10^−16^). Total X read numbers were summed at sex-linked SNP locations in each contig and normalized for each individual separately, then averaged among males and females to get the Xmale/2Xfemale ratio.

The observation that many X/Y pairs show reduced Y expression (Figure 1) raises the question whether dosage compensation has evolved in *S. latifolia*. To test this, we compared expression levels of sex-linked genes between males and females following a normalization procedure that allows comparing different individuals (see Material and Methods). First, we computed the ratio of the expression intensities of X-linked contigs in males and females and called this the Xmale/2Xfemale expression ratio (to stress the difference in gene copy number between male and female). In the absence of dosage compensation, the Xmale/2Xfemale expression ratio is expected to be 0.5, simply because males (XY) have one X-linked copy and females (XX) have two. This is what we observe for contigs that do not show reduced expression of the Y-linked allele relative to the X-linked allele, i.e., that have a Y/X expression ratio close to 1 (median of Xmale/2Xfemale ratio is 0.51 for contigs with 1≤Y/X<1.5; see Figure 2). However, for contigs with reduced Y expression and therefore low Y/X ratios, we observe an Xmale/2Xfemale expression ratio very close to 1 (median of contigs with Y/X<0.5 is 0.93; see Figure 2). This suggests that for contigs with reduced Y expression, for which expression of sex-linked genes would thus be unbalanced between males and females, a mechanism has evolved that compensates for the reduced Y expression by increasing X expression in males.

**Figure 3:**
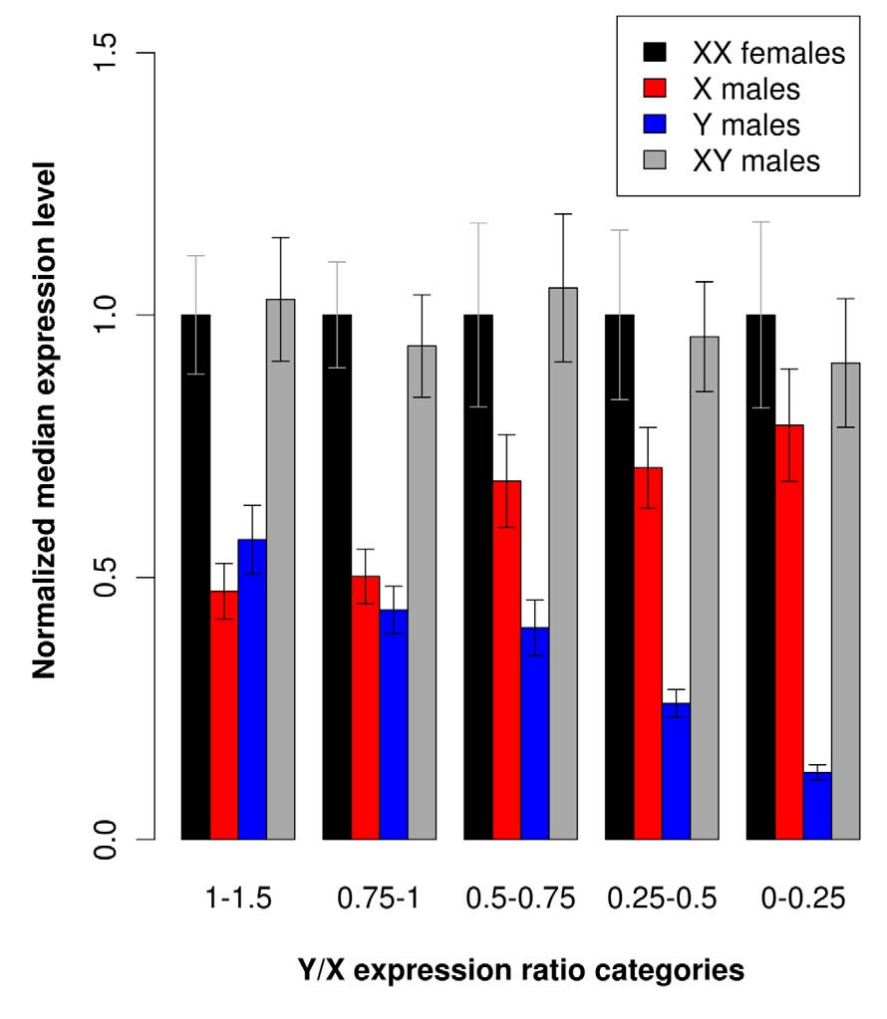
Expression levels of sex-linked contigs in both sexes for different Y/X expression ratio categories. Total read numbers were summed at sex-linked SNP locations and normalized for each individual and contig separately; medians for all contigs and individuals of the same sex were then obtained. Contigs with Y/X expression ratios above 1.5 were excluded, as well as contigs with Xmale/2Xfemale ratios above 2 (see text for explanations), which reduces the dataset to 1,346 sex-linked contigs. XX females, median expression level of both X-linked alleles in females; X males, median expression level of the single X-linked allele in males; Y males, median expression level of the Y-linked allele in males; XY males, median expression level of the X-linked plus Y-linked alleles in males. To compare different Y/X expression ratio categories, medians were normalized using the XX expression levels in females. Sample sizes are: 0–0.25, 110; 0.25–0.5, 269; 0.5–0.75, 315; 0.75–1, 341; 1–1.5, 315. Note that we do not have any contig with Y/X = 0 as our method did not allow us to detect such contigs (see Material and Methods). Error bars indicate 95% confidence intervals.

To study this phenomenon further, we compared expression of X-linked and Y-linked alleles in males and females for different Y/X expression ratio categories (Figure 3). We excluded sex-linked contigs that showed either an elevated Y expression (high Y/X ratios) or male-biased X expression (high Xmale/2Xfemale ratios). Such male-biased expression patterns suggest that these genes may be sexually antagonistic genes. The evolutionary dynamics of such genes is known to be distinct from other sex-linked genes and no dosage compensation is expected [48],[49]. Figure 3 shows the results for the remaining 75% of sex-linked genes. We found that X expression in males increases with decreasing Y expression, which results in similar expression levels of sex-linked contigs in both sexes and provides further evidence of dosage compensation in *S. latifolia*. Importantly, this result is consistent even when we include only sex-linked contigs with at least two sex-linked SNPs, for which we estimated the rate and number of erroneous sex-linked contigs to be extremely low (0.001 and 1.38, respectively; see Figure S4). We also looked at expression patterns of the contigs corresponding to known sex-linked genes. Although this analysis can only be qualitative due to the small number of such genes, we found that Y/X ratios for most genes are consistent with previous work [33] and some known sex-linked genes show evidence for dosage compensation (Table S4).

## Discussion

### Evidence for X Chromosome Dosage Compensation in *S. latifolia*

There was a recent claim of absence of dosage compensation in *S. latifolia* [42], which seems to contradict our findings. However, the test for dosage compensation performed in this recent work is very different from ours. As Chibalina and Filatov (2011) analyzed crosses (parents and progeny), they were able to identify X-linked genes without detectable homologous Y-linked copies (called hemizygous genes). They compared the expression levels of these hemizygous genes between sexes, found a significantly reduced expression in males compared to females, and concluded that this was evidence for the absence of dosage compensation in *S. latifolia* [42]. Their test however may be overly conservative, as it requires a strict Xmale/2Xfemale ratio of 1 to infer for dosage compensation. Their figure 4 suggests the Xmale/2Xfemale ratio is not 0.5, as expected under a complete absence of dosage compensation, but instead is close to 0.7, which is consistent with many hemizygous genes being dosage compensated. Importantly, the hemizygous genes were interpreted as sex-linked genes with fully degenerated Y copies, which may not always be the case as genes that have recently moved from the autosomes to the X chromosome will also be detected as hemizygous genes but dosage compensation is clearly not expected for those genes [43]. Such gene movement has been documented in *S. latifolia* [39] and may account for the intermediate Xmale/2Xfemale value (between 0.5 and 1) found in [42]. By contrast, we looked for departure from a Xmale/2Xfemale of 0.5 and did not restrict the test to sex-linked genes with no Y expression but included the many sex-linked genes with reduced but still detectable Y expression. We thus performed a more permissive test for dosage compensation, which may be more suitable in the case of young sex chromosomes with incipient X chromosome dosage compensation.

### Sex Bias in Gene Expression and Dosage Compensation

Dosage compensation is not the only sex-specific gene expression regulation that is expected on the X chromosome. Indeed, X-linked genes involved in sexual conflicts—for instance those underlying sexual dimorphism and having sexually antagonistic effects—can show sex-biased expression and this can substantially affect the global X expression pattern in both sexes if these genes are numerous [50]. A way to distinguish dosage compensation from such sex-specific expression regulation is to look at the X over autosome (X/A) expression ratio as only dosage compensation predicts a X/A expression of 1 [50]. However, this test is difficult to perform here for several reasons. First, our set of sex-linked genes is expected to exclude those with very low expression levels because the detection of sex-linked SNPs requires reasonably high read coverage. This should bias upward the average expression level of sex-linked genes compared to the “autosonal” set, which is what we actually found (the mean number of reads per base is 466.7 for sex-linked contigs and 101.4 for non–sex-linked contigs). Second, we do not have a reliable “autosomal” set as this includes a mixture of autosomal contigs and sex-linked contigs not detected by our method (∼40% of all sex-linked genes, see above). Although we excluded possible candidates for sexually antagonistic genes (some of the contigs with high Xmale/2Xfemale may be “male-beneficial and female-detrimental” genes), we cannot completely rule out the possibility that others remained in the set of contigs used to assess dosage compensation (especially some contigs with low Xmale/2Xfemale may be “female-beneficial and male-detrimental” genes). However, Figure 3 shows that the increase of X expression in males follows the level of degeneration of Y expression, which is not expected in case of sexually antagonistic selection. Moreover, increased expression of the X-linked allele in males always compensates for the reduced Y expression, such that the total expression of these sex-linked genes is similar in both sexes (i.e., X+Y expression in males = X+X expression in females), which is not in agreement with sexually antagonistic selection. On the contrary, sexually antagonistic selection predicts between-sex differences in expression of sex-linked genes. The results presented in Figure 3 are thus better explained by dosage compensation than by sexually antagonistic selection.

### Dosage Compensation in XY and ZW Systems

Global dosage compensation has previously been documented in male heterogametic systems (XY) such as *Drosophila*, *Caenorhabditis elegans*, and mammals [14],[51], whereas only partial (or no) dosage compensation has been found in female heterogametic systems (ZW) [52]. Indeed, in zebra finch, chicken, and crow, no global mechanism to balance avian Z chromosome gene dosage (such as X chromosome inactivation) has been found [53]–[56] and in chicken, dosage compensation seems to be local, with only few Z-linked genes being dosage compensated [57]. Similar observations have been made in silkworm [58],[59], indicating that the lepidopteran Z is not fully dosage compensated, and also in the parasite *Schistosoma mansoni* [60]. Moreover, studies on the platypus [61],[62] and on sticklebacks [63] suggest that partial dosage compensation can also exist in male heterogametic systems (XY). Overall, these new data suggest that full dosage compensation is not a necessary outcome of sex chromosome evolution [50]. An important point of whether dosage compensation will evolve or not is the presence of dosage-sensitive genes on the proto-sex chromosomes, as these genes are the only ones for which dosage compensation is vital [50],[64]. Although we do not have any data about the fraction of dosage-sensitive genes in the different sex chromosome systems, it has been suggested that resistance to aneuploidy and polyploidization may indicate whether the genome as a whole includes many such genes or not [50]. Polyploidization is known to be common in plants [65]. However, plant polyploids do have dosage problems that cause endosperm development failure and reduced fertility [64],[66]. Following polyploidization events, the retention of plant duplicate genes seems to be driven by dosage constraints as in animals [64]. All this suggests that the success of polyploids in plants may not be related to lack of dosage constraints but to other reasons (e.g., vegetative propagation). It is also known that aneuploidy has more severe phenotypic consequences than polyploidy in plants, which further supports the idea of strong dosage constraints in plant genomes [64]. As far as we know, there is no documented case of fertile polyploids in dioecious *Silene* species and it is possible that the *S. latifolia* genome includes enough dosage-sensitive genes for dosage compensation to evolve.

### Mechanisms of Dosage Compensation in Plants

Our results reveal that dosage compensation is not restricted to animals but also occurs in plants and raise questions about the mechanisms underlying dosage compensation. In animals, three different dosage compensation mechanisms have been uncovered (reviewed in [67]): hyper-expression of X-linked alleles in male *Drosophila*, down-regulation of the two X-linked alleles in hermaphrodites of *C. elegans*, and inactivation of one of the two female X chromosomes in mammals. We tested whether such a chromosome-wide inactivation exists in *S. latifolia* by checking whether both X-linked alleles are expressed in females. Although heterozygosity is low in our X-linked alleles because our individuals are inbred, we found that the level of heterozygosity of the X-linked alleles is similar for sex-linked contigs with dosage compensation and those without dosage compensation (Table S5). This suggests that both X-linked alleles are expressed, whatever the level of dosage compensation is, and does not support an X-inactivation-like mechanism in *S. latifolia*. Further work will be needed to identify the molecular mechanism underlying dosage compensation in *S. latifolia*.

### De Novo Evolution of Dosage Compensation in a Young XY System

Previous work in animals has reported dosage compensation in old X chromosomes (see above) and also in young neoX chromosomes such as the *D. miranda* neoX. The fusion between X and the autosome that formed the *D. miranda* neoX is very recent (1.5 million years old), but dosage compensation is achieved by a protein complex (the MSL complex) that pre-dates neoX formation and has been shown to be very old [68]. Evidence for de novo evolution of dosage compensation in evolutionary young animal sex chromosomes is therefore lacking [50]. In the *Silene* genus, most species are hermaphroditic or gynodioecious and do not have sex chromosomes. Sex chromosomes have evolved recently in two independent lineages, one including *S. latifolia* and one containing *S. colpophylla* [20],[44],[69]. Our results therefore reveal that dosage compensation has evolved de novo in evolutionarily young sex chromosomes in probably less than 10 million years. Furthermore, Figure 2 shows that many dosage-compensated contigs have an Xmale/2Xfemale ratio that is not exactly 1 (although the median is close to 1, there is no peak at 1 for Y/X<0.5 contigs). This is consistent with the mechanism being evolutionarily young and not optimized yet. Our results also reveal that dosage compensation can evolve as soon as Y expression starts declining. This way, dosage compensation already exists when the Y copy is ultimately lost (and can even facilitate such loss, see [70]). Instead of being a later step of sex chromosome evolution following Y degeneration, our results suggest that the evolution of dosage compensation and Y degeneration probably occur at the same time.

## Material and Methods

### Plant Material, RNA Extraction, Sequencing, and Assembly of Illumina Data

Plants used in this study belong to a population of *S. latifolia* that has been inbred for ten generations with brother-sister mating: three males (U10_11, U10_49, and U10_09) and three females (U10_34, U10_37, and U10_39) that were grown in a temperature-controlled greenhouse. The QiagenRNeasy Mini Plant extraction kit was used to extract total RNA two times separately from four flower buds at developmental stages B1–B2 after removing the calyx. Samples were treated additionally with QiagenDNase. RNA quality was assessed with an Aligent Bioanalyzer (RIN>9) and quantity with an Invitrogen Qubit. An intron-spanning PCR product was checked on an agarose gel to exclude the possibility of genomic DNA contamination. Then, the two extractions of the same individual were pooled. Samples were sequenced by FASTERIS SA on an Illumina HiSeq2000 following an Illumina paired-end protocol (fragment lengths 150–250 bp, 100 bp sequenced from each end). Individuals were tagged and pooled for sequencing in two different runs (U10_49 male and U10_37 female in the first run and the others in the second). See Table S1 for sizes of the different libraries. Our Illumina reads are available in the GEO database (through the GEO Series GSE35563).

De novo assembly was conducted on a computer cluster (Figure S1). Illumina reads from all individuals were pooled together for assembly with AbySS 1.2.5 (*E* = 10, *n* = 5) [71] with the paired-end option and with all k-mers ranging from 51 to 96 in order to address variable transcript expression [72]. A k-mer length equal to 51 was the minimum possible to avoid contigs shorter than the reads, and 96 is the maximum allowed by AbySS. Only contigs were kept at this stage, singlets were discarded. Contigs that exactly matched another longer contig were then removed by pairwise comparison of AbySS outputs using Trans-ABySS 1.2.0 [72]. A non-redundant set of contigs was thus obtained and further assembled through two runs of CAP3 version 12/21/07 [73]. Singlets and contigs were conserved after each CAP3 run. CAP3 runs increased the chance for X and Y copies to be assembled into the same contig, which is crucial for further sex-linked SNP detection. Contigs shorter than 200 bp were not included in the final set of contigs.

### Mapping, SNPs Analysis, and Sex-Linkage Detection

Illumina reads were mapped onto reference sequences (final set of contigs and also CDS from known sex-linked genes retrieved from GenBank for adjusting SNP detection, see below) for each individual separately using BWA 0.5.9 [74] (using default parameters for paired-end reads, and gap and mismatch maximum number of 5 as suggested for 100 bp reads in [74]), which was shown to be efficient and to use much less RAM than other programs for Illumina read mapping [75]. Alignments of all individuals were then merged together using Samtoolsmerge version 0.1.12 [76]. The percentage of mapped reads was assessed using Samtoolsflagstat version 0.1.12 [76] and the average coverage was determined using the Genome Analysis Toolkit (GATK 1.0.5315) Depth of Coverage [77].

SNPs were detected with the GATK Unified Genotyper (using the following parameters: -stand_call_conf 4 -stand_emit_conf 0 -mbq 17 -mmq 0 -mm40 40 -bad_mates -dcov 2000) [77], which is considered the best currently available tool for SNP detection [78]. Thresholds for the different SNP detection parameters were set to be very low (except for the base quality parameter) in order not to disfavour Y SNPs that are expected to be found in low numbers and low mapping quality if a contig contains mainly X reads, which can happen when X-linked alleles are more strongly expressed than Y-linked alleles [33].

The detected SNPs were then filtered using Perl scripts to retrieve SNPs for which all males are heterozygous (XY) and all females homozygous (XX). All contigs with at least one SNP showing this pattern were considered sex-linked. For females, the genotypes inferred by GATK were directly used for analysis. For males, this information is not reliable since the Y-linked allele is expected to be less expressed than the X-linked allele [33] while GATK genotyper makes the assumption that both alleles are expressed at a similar level. The read numbers of each SNP were thus used to infer male genotypes (see Text S3 for details).

Polymorphism on the X chromosome (at least one male or female heterozygous or all individuals homozygous but not for the same polymorphism) was detected on sex-linked contigs with a similar filter as the one described above.

### Estimates of Expression Levels of the Sex-Linked Contigs

Expression levels of the X-linked and Y-linked alleles in males and both X copies in females were computed by counting reads at sex-linked SNP locations only, and not for the entire contigs, in order to clearly distinguish between X and Y reads. Total read numbers of all X or Y SNPs provided by the GATK Unified Genotyper [77] were summed for each X-linked or Y-linked alleles and each individual separately and then normalized using the total number of mapped reads per individuals (library size) and the number of sex-linked SNPs in the contigs:
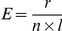
With *E* = normalized expression level, *r* = sum of total read counts, *n* = *n* sex-linked SNPs, *l* = normalized library size.

The library size of the six individuals was normalized to take into account the difference in mitochondrial, chloroplast, and transposable element (TE) transcript quantity between sexes and the difference in rRNA quantity between the first and the second Illumina run. The *Arabidopsis thaliana* rRNA genes, complete *S. latifolia* mtDNA genome [79], *S. latifolia* chloroplast genes *rpoB*, *rpoC1*, *rpoC2*, *rps2*, *atpI*, *atpH*, *atpF*, *atpA*, *psbI*, *psbK*, *rps16*, *matK*, *psbA*, *rpl2*, *ycf2*, *ndhB*, *rps7*, and the TEs known in *Silene* [80] were retrieved from GenBank. The read numbers of rRNA, TEs and mtRNA, and cpRNA were determined by mapping the Illumina reads onto the known CDS sequences of these elements using the default parameters in BWA (results presented in Table S1).

The expression levels were normalized for each contig and for each individual in number of reads per kilobase per million mapped reads (RPKM) [81], and then the mean for each sex was computed.

## Supporting Information

Figure S1 **Assembly, mapping, and SNP analysis.** Steps of the de novo assembly. From left to right: during first assembly with ABySS, k-mers ranging from 51 from 96, only contigs were kept. Pairwise comparisons of contigs were then done by Trans-ABySS in order to remove small contigs that exactly matched longer contigs. Contigs were then further assembled by two runs of CAP3 (mismatches and partial overlaps allowed); singlets and contigs were kept after each run. Illumina reads were mapped onto the contigs with BWA and SNPs were detected with GATK. SNPs were then analyzed in order to detect sex-linked SNPs (all males heterozygous XY, and all females homozygous XX).
(TIFF)

Figure S2 **Number of sex-linked SNPs detected and coverage for known sex-linked genes.** cDNA sequences of previously identified sex-linked genes were retrieved from GenBank. Illumina reads were mapped on the cDNA sequences using BWA and SNP detection was done as in Material and Methods. We then computed the number of sex-linked SNPs detected over the number of known sex-linked SNPs for these genes and compared this with the number of reads ( = coverage) for each X/Y gene pairs. Sex-linked genes were grouped by strata as in [82].
(TIFF)

Figure S3 **Size (bp) distribution of sex-linked contigs.**
(TIFF)

Figure S4 **Expression levels of sex-linked contigs in both genders for different Y/X expression ratio categories for contigs with ≥2 sex-linked SNPs (1,009 contigs).** The legend is the same as for Figure 3 except for contig numbers: 0–0.25, 66; 0.25–0.5, 165; 0.5–0.75, 248; 0.75–1, 279; 1–1.5, 251.
(TIFF)

Table S1 **Raw Illumina data and results of the assembly.**
(DOC)

Table S2 **Contig statistics.**
(DOC)

Table S3 **Results of SNP analysis for known autosomal and sex-linked genes.**
(RTF)

Table S4 **Analysis of expression patterns in known sex-linked genes.**
(RTF)

Table S5 **Levels of heterozygosity of the X-linked alleles with and without dosage compensation.**
(DOC)

Text S1 **Identification and validation of new sex-linked genes.**
(RTF)

Text S2 **Simulations to estimate the rate of false positive sex-linked genes.**
(DOC)

Text S3 **SNP detection and filtering.**
(DOC)
