# Supplementary figures and images for: Rapid De Novo Evolution of X Chromosome Dosage Compensation in Silene latifolia, a Plant with Young Sex Chromosomes
Source: PLoS Biol. 2012 Apr 17;10(4):e1001308. doi: 10.1371/journal.pbio.1001308 (PMC3328428; doi:10.1371/journal.pbio.1001308)

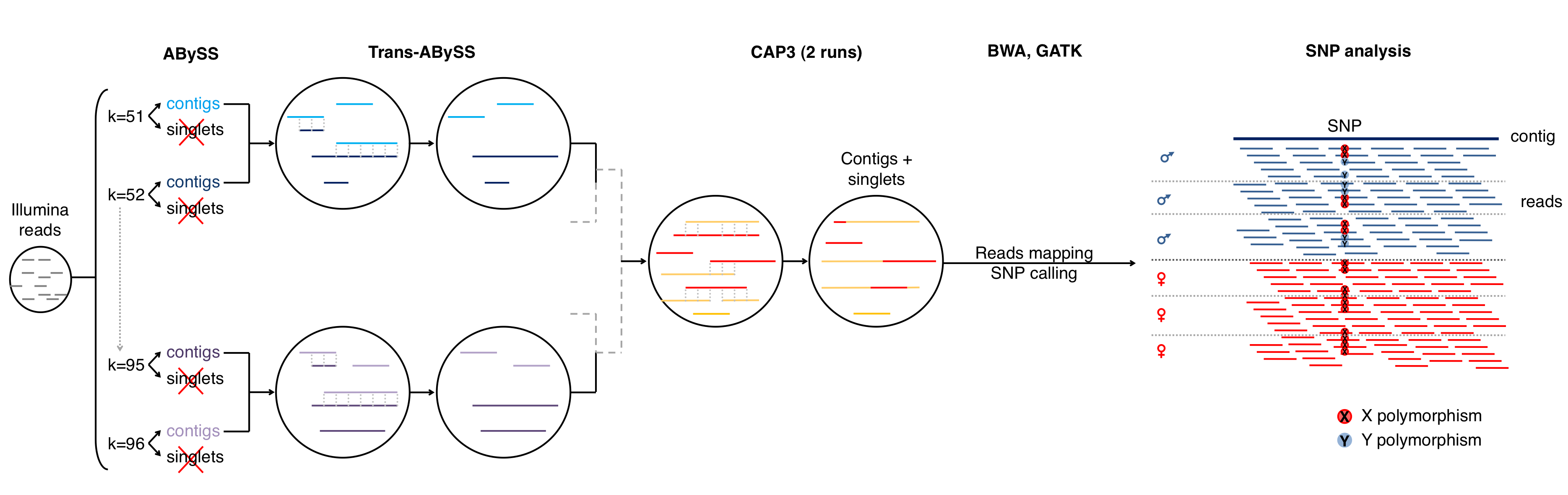

Supplement: Figure S1 — Assembly, mapping, and SNP analysis. Steps of the de novo assembly. From left to right: during first assembly with ABySS, k-mers ranging from 51 from 96, only contigs were kept. Pairwise comparisons of contigs were then done by Trans-ABySS in order to remove small contigs that exactly matched longer contigs. Contigs were then further assembled by two runs of CAP3 (mismatches and partial overlaps allowed); singlets and contigs were kept after each run. Illumina reads were mapped onto the contigs with BWA and SNPs were detected with GATK. SNPs were then analyzed in order to detect sex-linked SNPs (all males heterozygous XY, and all females homozygous XX). (TIFF) [file pbio.1001308.s001.tif]

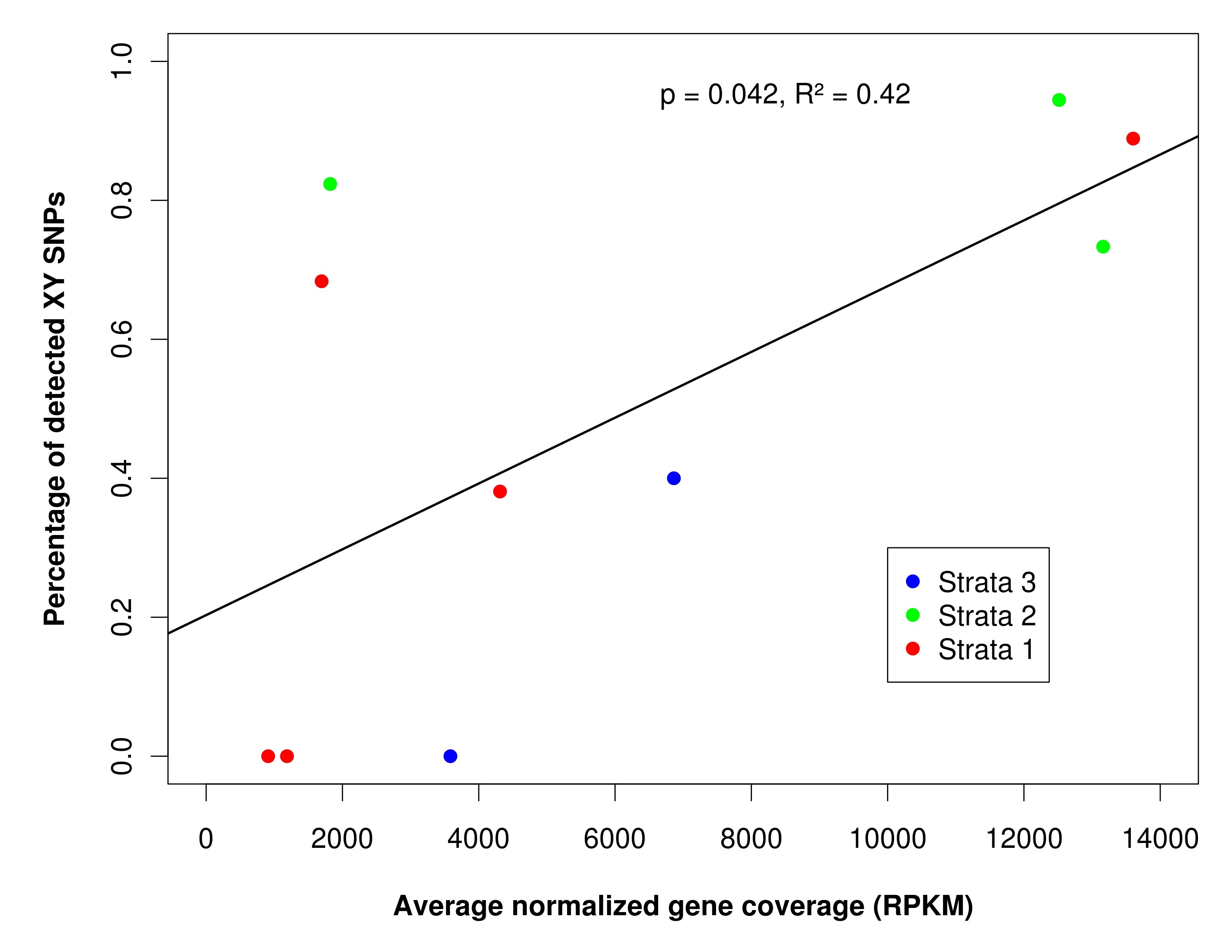

Supplement: Figure S2 — Number of sex-linked SNPs detected and coverage for known sex-linked genes. cDNA sequences of previously identified sex-linked genes were retrieved from GenBank. Illumina reads were mapped on the cDNA sequences using BWA and SNP detection was done as in Material and Methods. We then computed the number of sex-linked SNPs detected over the number of known sex-linked SNPs for these genes and compared this with the number of reads ( = coverage) for each X/Y gene pairs. Sex-linked genes were grouped by strata as in [82]. (TIFF) [file pbio.1001308.s002.tif]

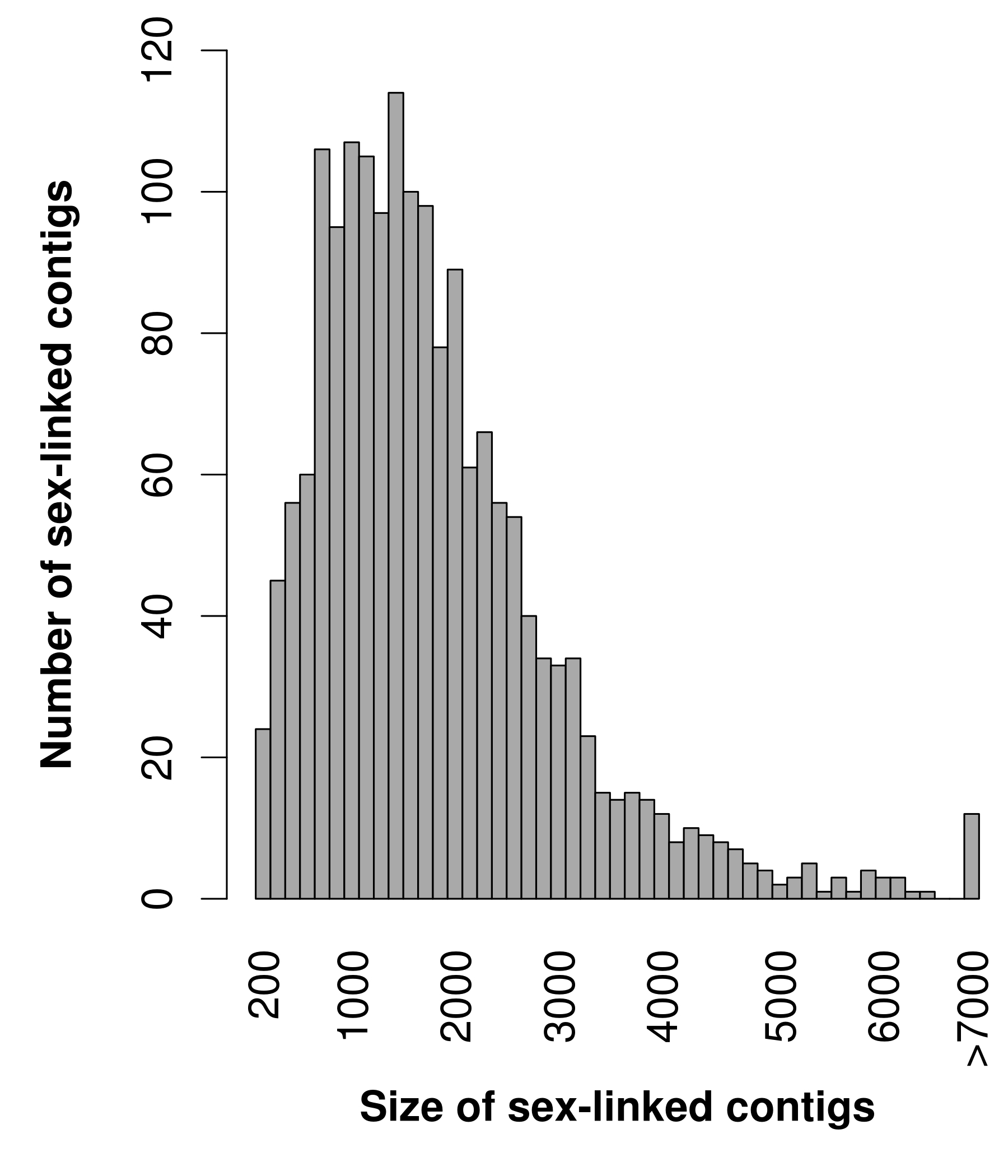

Supplement: Figure S3 — Size (bp) distribution of sex-linked contigs. (TIFF) [file pbio.1001308.s003.tif]

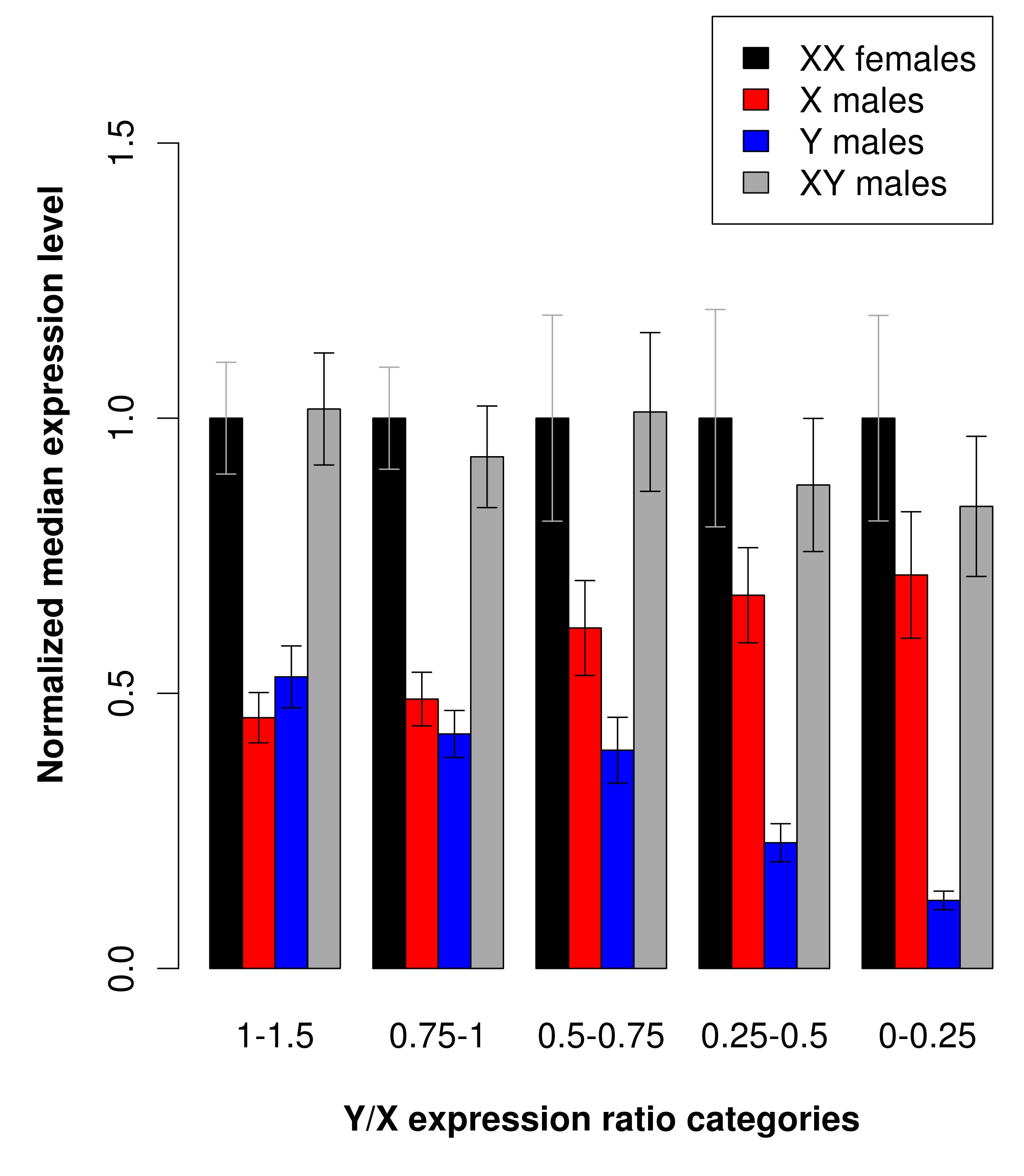

Supplement: Figure S4 — Expression levels of sex-linked contigs in both genders for different Y/X expression ratio categories for contigs with ≥2 sex-linked SNPs (1,009 contigs). The legend is the same as for Figure 3 except for contig numbers: 0–0.25, 66; 0.25–0.5, 165; 0.5–0.75, 248; 0.75–1, 279; 1–1.5, 251. (TIFF) [file pbio.1001308.s004.tif]
