# Supplementary material for: Rapid De Novo Evolution of X Chromosome Dosage Compensation in Silene latifolia, a Plant with Young Sex Chromosomes
Source: PLoS Biol. 2012 Apr 17;10(4):e1001308. doi: 10.1371/journal.pbio.1001308 (PMC3328428; doi:10.1371/journal.pbio.1001308)
Supplement: Table S1 — Raw Illumina data and results of the assembly. (DOC) [file pbio.1001308.s005.doc]

**Table S1. Raw Illumina data and results of the assembly.** Size of the dataset (numbers of reads and numbers of bases), numbers of mapped reads, coverage and normalized library size for each individual. The coverage is the average number of reads per base and the normalized library size corresponds to the number of mapped reads minus the number of reads that belong to rRNA, TEs, cpRNA, mtRNA (see Material and Methods). The normalized library sizes were used to compute normalized expression levels.

| **Individual** | **U10_37 (female)** | **U10_49 (male)** | **U10_11 (male)** | **U10_9 (male)** | **U10_39 (female)** | **U10_34 (female)** | **Total** |
| --- | --- | --- | --- | --- | --- | --- | --- |
| **Number of reads** | 75800468 | 89940748 | 48720356 | 46671858 | 44704710 | 45662378 | 351500518 |
| **Number of bases (Gb)** | 7.58 | 8.99 | 4.87 | 4.67 | 4.47 | 4.57 | 35.15 |
| **Number of mapped reads** | 66368044 | 80010603 | 45966660 | 44133217 | 41605007 | 43128090 | 321211621 |
| **Coverage** | 29.96 | 39.22 | 33.98 | 33.09 | 32 | 33.03 | 201.3 |
| **Normalized library size** | 58048299 | 73475899 | 45617786 | 43766170 | 41190095 | 42773518 | 304871767 |
