# Supplementary material for: Rapid De Novo Evolution of X Chromosome Dosage Compensation in Silene latifolia, a Plant with Young Sex Chromosomes
Source: PLoS Biol. 2012 Apr 17;10(4):e1001308. doi: 10.1371/journal.pbio.1001308 (PMC3328428; doi:10.1371/journal.pbio.1001308)
Supplement: Table S2 — Contig statistics. (DOC) [file pbio.1001308.s006.doc]

**Table S2. Contig statistics**.

|  | **Number of contigs** | **Mean size (bp)** | **Median size (bp)** | **Max size (bp)** | **Min size (bp)** | **Mean coverage (X)** | **Mean SNP # / contig** |
| --- | --- | --- | --- | --- | --- | --- | --- |
| **All contigs** | 141,855 | 748.5 | 410 | 20,988 | 200 | 201.3 | 10.26 |
| **Sex-linked contigs** | 1736 | 1931 | 1673 | 16,746 | 207 | 427.2 | 22.97 |
