# Supplementary material for: Rapid De Novo Evolution of X Chromosome Dosage Compensation in Silene latifolia, a Plant with Young Sex Chromosomes
Source: PLoS Biol. 2012 Apr 17;10(4):e1001308. doi: 10.1371/journal.pbio.1001308 (PMC3328428; doi:10.1371/journal.pbio.1001308)
Supplement: Table S3 — Results of SNP analysis for known autosomal and sex-linked genes. (RTF) [file pbio.1001308.s007.rtf]

Table S3. Results of SNP analysis for known autosomal and sex-linked genes. Numbers of sex-linked SNPs identified for known autosomal and sex-linked genes in S. latifolia. The cDNA sequences retrieved from GenBank were blasted onto the final contig set of the assembly to identify the corresponding contig of each gene and SNPs were studied to retrieve those with a sex-linked pattern. Autosomal cDNA sequences are from [44]. Sex-linked cDNA sequences are from GenBank.

Genes	Number of sex-linked SNPs	Type	References	
SlXY7	38	Sex-linked gene	[25]	
SlXY1	5	Sex-linked gene	[35]	
SlXY9	6	Sex-linked gene	[40]	
SlMF1 SlMF1 did not have sufficient coverage (it is a very weakly expressed gene) to study polymorphisms and thus could not be identified as sex-linked.	0	Sex-linked gene	[90]	
SlssXY	4	Sex-linked gene	[38]	
SlCypXY	38	Sex-linked gene	[25]	
DD44XY	26	Sex-linked gene	[37]	
SlXY3	2	Sex-linked gene	[21]	
SlAP3XY SlAP3XY and SlXY4 could not be detected as sex-linked because their X and Y copies were assembled into different contigs, which is not surprising, given that these genes have highly divergent X/Y copies [21,25].	0	Sex-linked gene	[91,92]	
SlXY42	0	Sex-linked gene	[36]	
SlX6a SlX6a and SlX6b were not identified as sex-linked because they are duplicate genes and were assembled into the same contig, which prevented detection of XY SNPs.	0	Sex-linked gene	[25]	
SlX6b3	0	Sex-linked gene	[25]	
2A10	0	Autosomal gene	[44]	
ABCtr	0	Autosomal gene	[44]	
ADPGph	0	Autosomal gene	[44]	
ATUB-A	0	Autosomal gene	[44]	
ClpP3	0	Autosomal gene	[44]	
ELF	0	Autosomal gene	[44]	
LIP21	0	Autosomal gene	[44]	
OxRZn	0	Autosomal gene	[44]	
PGK	0	Autosomal gene	[44]	
PSIcentII	0	Autosomal gene	[44]	

Additional references
90. Matsunaga S, Lebel-Hardenack S, Kejnovsky E, Vyskot B, Grant SR, et al. (2005) An anther- and petal-specific gene SlMF1 is a multicopy gene with homologous sequences on sex chromosomes. Genes Genet Syst 80: 395-401.
91. Matsunaga S, Isono E, Kejnovsky E, Vyskot B, Dolezel J, et al. (2003) Duplicative transfer of a MADS box gene to a plant Y chromosome. Mol Biol Evol 20: 1062-1069.
92. Cegan R, Marais GA, Kubekova H, Blavet N, Widmer A, et al. (2010) Structure and evolution of Apetala3, a sex-linked gene in Silene latifolia. BMC Plant Biol 10: 180.
