# Supplementary material for: Rapid De Novo Evolution of X Chromosome Dosage Compensation in Silene latifolia, a Plant with Young Sex Chromosomes
Source: PLoS Biol. 2012 Apr 17;10(4):e1001308. doi: 10.1371/journal.pbio.1001308 (PMC3328428; doi:10.1371/journal.pbio.1001308)
Supplement: Table S4 — Analysis of expression patterns in known sex-linked genes. (RTF) [file pbio.1001308.s008.rtf]

Table S4. Analysis of expression patterns in known sex-linked genes. These sex-linked genes have been identified in previous work (see Table S3 for references). Data are not available for SlMF1, SlX6a and SlX6b (see Table S3 for more details) and these genes are not included in the table. Y/X ratios from this study and from experimental data [33] are well correlated (except for SlCypXY). We computed the ratio of male expression (X + Y) over female expression (both X-linked copies). In absence of dosage compensation (DC), this ratio should be 1 for genes without Y degeneration (Y/X ratio ~ 1) and 0.5 for genes with Y degeneration (low Y/X ratios). In presence of DC, this ratio should be 1 for both genes without Y degeneration (Y/X ratio ~ 1) and with Y degeneration (low Y/X ratios) as in Figure 3. Here we considered that genes with high Y/X ratios do not need DC, genes with low Y/X ratios and male expression (X + Y) over female expression (both X-linked copies) ratios >0.8 and <1.2 are consistent with DC. Other genes are considered equivocal.

Genes	Y/X ratio (Illumina data)	Y/X ratio (experimental data)	Male exp (X+Y) / Female exp (2X)	Observations	
SlXY3	0.06	0.56	1.70	Equivocal	
SlXY7	0.13	0.32	0.87	Consistent with DC DC = dosage compensation	
SlXY42	0.45	0.33	0.89	Consistent with DC	
SlXY9	0.49	na	0.66	Equivocal	
DD44XY	0.61	0.59	0.95	Consistent with DC	
SlAP3XY SlAP3XY and SlXY4 are not among the sex-linked contigs that we identified because X and Y copies are too divergent and were assembled independently (see Table S3). We used SlAP3X and SlX4 from Genbank to map X and Y Illumina reads and estimate expression.	0.62	na na = not available.	1.78	Equivocal	
SlssXY	0.70	na	1.01	DC not needed	
SlXY1	0.83	1.13	0.59	DC not needed	
SlCypXY	1.07	0.14	0.68	DC not needed	
