# Supplementary material for: Rapid De Novo Evolution of X Chromosome Dosage Compensation in Silene latifolia, a Plant with Young Sex Chromosomes
Source: PLoS Biol. 2012 Apr 17;10(4):e1001308. doi: 10.1371/journal.pbio.1001308 (PMC3328428; doi:10.1371/journal.pbio.1001308)
Supplement: Table S5 — Levels of heterozygosity of the X-linked alleles with and without dosage compensation. (DOC) [file pbio.1001308.s009.doc]

**Table S5. Levels of heterozygosity of the X-linked alleles with and without dosage compensation.** The level of heterozygosity (number of heterozygous sites/bp) was computed for each Y/X expression ratio category. The fraction of polymorphic sites can vary from one category to another and we also computed the fraction of heterozygous SNPs among the sex-linked SNPs for each Y/X expression ratio category. Only sex-linked contigs in low Y/X ratio categories show dosage compensation. Results are shown for each female individually and averaged for all females.

| **Y/X ratio** | **< 0.25** | **0.25 - 0.5** | **0.5 - 0.75** | **0.75 - 1** | **1 - 1.5** | **≥ 1.5** |
| --- | --- | --- | --- | --- | --- | --- |
| **Level of heterozygosity for female U10_34** | 0.0059 | 0.0054 | 0.0034 | 0.0038 | 0.0035 | 0.0029 |
| **Level of heterozygosity for female U10_37** | 0.0054 | 0.0050 | 0.0033 | 0.0036 | 0.0034 | 0.0030 |
| **Level of heterozygosity for female U10_39** | 0.0057 | 0.0055 | 0.0035 | 0.0038 | 0.0036 | 0.0031 |
| **Average level of heterozygosity** | 0.0057 | 0.0053 | 0.0034 | 0.0037 | 0.0035 | 0.0030 |
|  |  |  |  |  |  |  |
| **% of heterozygous SNPs for female U10_34** | 44% | 42% | 30% | 32% | 29% | 32% |
| **% of heterozygous SNPs for female U10_37** | 41% | 39% | 28% | 30% | 28% | 33% |
| **% of heterozygous SNPs for female U10_39** | 43% | 43% | 30% | 32% | 30% | 34% |
| **Average % of heterozygous SNPs** | 43% | 42% | 29% | 31% | 29% | 33% |
