# Supplementary material for: Rapid De Novo Evolution of X Chromosome Dosage Compensation in Silene latifolia, a Plant with Young Sex Chromosomes
Source: PLoS Biol. 2012 Apr 17;10(4):e1001308. doi: 10.1371/journal.pbio.1001308 (PMC3328428; doi:10.1371/journal.pbio.1001308)
Supplement: Text S1 — Identification and validation of new sex-linked genes. (RTF) [file pbio.1001308.s010.rtf]

Text S1. Identification and validation of new sex-linked genes

Identification of new sex-linked genes
The S. latifolia haploid genome has been estimated to be around 3 Gb and to have a high DNA repeat content [23]. Because the genome is complex and has not been sequenced, we used RNA-seq -a next-generation transcriptome-sequencing approach- that is increasingly being used in non-laboratory organisms to identify new genes [83,84,85] and is ideally suited to study gene expression including that of sex-linked genes [51,86,87]. We used 3 males and 3 females from a S. latifolia population that has been inbred for 10 generations. For each individual, RNA extracted from flower buds was tagged and sequenced using Illumina paired-end technology (2 x 100 bp) for ~35 Gb of sequences in total (Table S1). Male and female reads were pooled and assembled de novo together using a pipeline (described in Material and Methods and Figure S1) and we obtained 141,855 contigs with a mean size of 748.5 bp (Table S2). From these, we identified sex-linked contigs using segregation analysis. 
The S. latifolia X and Y chromosomes are expected to include several gene categories: many (if not most) sex-linked genes should derive from the original autosomal pair that gave rise to S. latifolia X and Y chromosomes [21,22,25], the other genes resulting from translocation from autosomes to the sex chromosomes (one such case is known in S. latifolia, see [39]) or from chromosome-specific duplications. How many of the S. latifolia sex-linked genes (of autosomal origin) have lost their Y copy following X-Y recombination suppression is currently unknown. However, given that S. latifolia sex chromosomes have originated recently, we expect many of these to have a functional Y-linked copy or at least a recognizable pseudogenized Y-linked copy, as has been found for the MROS3 gene [26]. We thus expect many of the sex-linked genes to have both an X-linked and a Y-linked copy. These genes are sometimes referred to as gametologs (= homologs arising through lack of recombination and subsequent differentiation of sex chromosomes, see [88]). Here we will use the terms X-linked and Y-linked alleles for convenience as our method relies on Single Nucleotide Polymorphism (SNP) detection (see below), although the synonymous divergence between these “alleles” ranges from 5-20% [21,22,25] and thus can be significantly higher than the level of polymorphism among autosomal alleles. The overall X-Y divergence (including both synonymous and non-synonymous sites) is, however, low in all known sex-linked genes, the X-linked and Y-linked alleles should thus assemble into a single contig for most sex-linked genes, with perhaps a decreased probability when the synonymous X-Y divergence is close to the upper limit detected so far (20%) and when X-Y divergence is notable at the non-synonymous level. We thus mapped all Illumina reads back onto the contigs (91.4% were successfully mapped, see Table S1) and searched for SNPs in the read alignments (see Material and Methods). A subset of SNPs showed patterns typical of sex-linkage (heterozygous in males and homozygous in females). This way, we found 16,308 sex-linked SNPs in 1736 contigs with at least one sex-linked SNP (Table S2). Using a similar approach, two recent studies found ~400 [42] and ~1800 [43] sex-linked genes. Differences in RNA-seq assembly and SNP filtering procedures may explain those different numbers. However, our estimate falls within the range of values from this recent work, and all studies do not differ greatly.

Validation of the new sex-linked genes
Establishing sex-linkage usually requires more than 6 individuals. However, we here used individuals from a highly inbred line whose level of polymorphism is expected to be very low. Indeed, the estimate for the level of polymorphism in our inbred line (= total number of detected SNPs divided by the total number of bp, taking all contigs into account) is 0.0074, and is thus about one order of magnitude lower than what has been reported for autosomal or sex-linked loci in natural populations of S. latifolia [30,34]. We thus expect SNPs in our contigs to arise mainly because of assembling together X-linked and Y-linked alleles, or recently duplicated genes, but only the former should show sex-linkage patterns. We nevertheless tested the reliability of our inference of sex-linkage in several ways. First, we used known autosomal genes [44] to see whether sex-linked SNPs have been wrongly inferred for these, but could not find any for the 10 autosomal genes tested (Table S3). Second, we simulated genotypes for 3 males and 3 females for ~40,000 autosomal SNPs using information on polymorphism level and sequencing errors from our data to check whether a pattern of sex-linkage could be obtained by chance for autosomal SNPs (Text S2). We found the rate of false sex-linked contigs to be very low (0.02) and as few as 37 sex-linked contigs may be erroneous. We therefore concluded that our inferences of sex-linkage are highly reliable. This is consistent with the very low rates of false positives found by testing experimentally 10 to 18 new sex-linked genes in the recent work of [42,43].
To estimate how many sex-linked contigs we missed with our method, we checked how many of the previously identified sex-linked genes (see list and references in Table S3) were among our sex-linked contigs. 42% of these were not found in our set of sex-linked contigs. Further analyses showed, as expected, that our method is less efficient when the X-linked and/or the Y-linked allele have low expression and thus low coverage, because this makes SNP detection unlikely (Figure S2) or when X-linked and Y-linked alleles are too divergent and are not combined into one single contig but instead into two distinct contigs (see Table S3 footnotes), which prevents inference of sex linkage. The actual number of sex-linked genes in S. latifolia could be ~4000 [43]. Part of the X is pseudoautosomal and still recombines with the Y during male meiosis. Genetic data suggest that the pseudoautosomal region (PAR) may be as large as 30 cM [89] and makes up as much as 1/3 of the X chromosome if we make the very simplistic assumption of similar recombination rates along the X chromosome. Only ~2600 putative genes on the X should then be located in the non-recombining region and could then be identified as sex-linked with our segregation analysis. Given our rate of missed sex-linked genes (see above), we expect to miss ~40% of the true sex-linked genes (i.e ~ 1040). This is consistent with the fact that about half of the X-specific region harbours genes with maximum (20%) X-Y synonymous divergence, which our method is likely to miss. This also means that we expect to find ~1560 sex-linked genes with our method. This figure relies on a very rough idea of how many sex-liked genes there could be in S. latifolia [43], but is nevertheless consistent with the actual number of sex-linked contigs that we found. Of course, some of our contigs may be fragments of the same transcripts. However, the size distribution of contigs (Figure S3) suggests that many of them are full-length transcripts as expected given the average coverage that we have here (~400X, see Table S2). 


Additional references
83. Vera JC, Wheat CW, Fescemyer HW, Frilander MJ, Crawford DL, et al. (2008) Rapid transcriptome characterization for a nonmodel organism using 454 pyrosequencing. Mol Ecol 17: 1636-1647.
84. Gibbons JG, Janson EM, Hittinger CT, Johnston M, Abbot P, et al. (2009) Benchmarking next-generation transcriptome sequencing for functional and evolutionary genomics. Mol Biol Evol 26: 2731-2744.
85. Brautigam A, Gowik U (2010) What can next generation sequencing do for you? Next generation sequencing as a valuable tool in plant research. Plant Biol (Stuttg) 12: 831-841.
86. Xiong Y, Chen X, Chen Z, Wang X, Shi S, et al. (2010) RNA sequencing shows no dosage compensation of the active X-chromosome. Nat Genet 42: 1043-1047.
87. Lott SE, Villalta JE, Schroth GP, Luo S, Tonkin LA, et al. (2011) Noncanonical compensation of zygotic X transcription in early Drosophila melanogaster development revealed through single-embryo RNA-seq. PLoS Biol 9: e1000590.
88. Garcia-Moreno J, Mindell DP (2000) Rooting a phylogeny with homologous genes on opposite sex chromosomes (gametologs): a case study using avian CHD. Mol Biol Evol 17: 1826-1832.
89. Bergero R, Charlesworth D personal communication.
