# Supplementary material for: Rapid De Novo Evolution of X Chromosome Dosage Compensation in Silene latifolia, a Plant with Young Sex Chromosomes
Source: PLoS Biol. 2012 Apr 17;10(4):e1001308. doi: 10.1371/journal.pbio.1001308 (PMC3328428; doi:10.1371/journal.pbio.1001308)
Supplement: Text S2 — Simulations to estimate the rate of false positive sex-linked genes. (DOC) [file pbio.1001308.s011.doc]

**Text S2. Simulations to estimate the rate of false-positive sex-linked contigs**

We ran simulations in order to estimate the rate of wrongly inferred sex-linked contigs. The idea is to simulate autosomal SNPs, then to run our procedure for detecting sex-linked SNPs (see Material and Methods and Text S3) on these simulated SNPs, and to check how many SNPs are inferred as being sex-linked when none are expected, which will give the rate of false positives sex-linked SNPs (and false positive sex-linked contigs). We did not use non-sex-linked contigs since this includes a mixture of autosomal contigs and sex-linked contigs undetected by our method and is not a true autosomal dataset. To be as close as the real data as possible, our strategy was to simulate autosomal SNPs with characteristics similar to the SNPs on the sex-linked contigs that we detected. To do this, we simulated autosomal SNPs using all 39,569 polymorphisms in our 1736 sex-linked contigs (sex-linked plus other SNPs) and sequencing errors data provided by FASTERIS.

In order to simulate autosomal SNPs, we used female genotypes as X and autosomes have similar levels of polymorphism in *S. latifolia* [34]. We computed the percentage of each genotypes in females using all SNPs from sex-linked contigs (% of females homozygous for the reference allele, % of females homozygous the alternative allele, % of heterozygous females). Six genotypes were randomly sampled from these genotype frequencies and assigned to three males and three females. For each individual, read numbers were obtained from the observed numbers at real SNPs from sex-linked contigs (all 39,569 SNPs were used one after the other). This means that our set of simulated autosomal SNPs takes into account the differences in expression levels that we observe between males and females and also between contigs (some being lowly expressed and other highly expressed). For heterozygous genotypes, read numbers were drawn from a binomial distribution with each polymorphism having an equal probability to be drawn, assuming they are equally expressed because autosomal. We then added sequencing errors by randomly exchanging alleles among each other using the PhiX error rates provided by FASTERIS. We thus obtained 39,569 simulated autosomal SNPs with, for each individual, the genotype and the read numbers of each allele.

Then, the scripts used to detect XY polymorphisms were run on the simulated autosomal SNP data and the proportion of false sex-linked contigs was computed. The proportion *α* of false XY SNPs observed in the simulated SNP data was used to compute the probability *P_i_* for each sex-linked contig *i* to be a false positive, given *n_i_* the number of XY SNPs of the contig and *m_i_* the other SNPs:

(1)

This probability was computed for each identified sex-linked contig and will depend on how polymorphic a contig is (accounting for differences in polymorphism levels between contigs). The expected number of false sex-linked contigs *F* is the sum of probabilities for each contig to be false:

(2)
