# Supplementary material for: Rapid De Novo Evolution of X Chromosome Dosage Compensation in Silene latifolia, a Plant with Young Sex Chromosomes
Source: PLoS Biol. 2012 Apr 17;10(4):e1001308. doi: 10.1371/journal.pbio.1001308 (PMC3328428; doi:10.1371/journal.pbio.1001308)
Supplement: Text S3 — SNP detection and filtering. (DOC) [file pbio.1001308.s012.doc]

**Text S3. SNP detection and filtering**

The empirical filter that follows was established after running GATK on known XY genes for training. At least 3 reads of good quality of each polymorphism were required for a male to be considered heterozygous. In case of a highly expressed gene (more than 1000 reads), the threshold was at least 10 reads of good quality for each polymorphism. If one male had 3 or less than 3 reads of good quality we could not infer its genotype reliably but if the other male and female individuals showed a clear sex-linked pattern, the SNP was considered XY all the same. In some cases, homozygous females had a few reads with the Y polymorphism (this observation was also made on known XY genes and can be attributed to sequencing or tag assignment errors). In such cases, the maximum number of reads observed with the Y polymorphism in females was used as a threshold to infer whether males were heterozygous. This was set to 5X/5Y for 1 Y read in females (20.1% of sex-linked SNPs) and respectively 20X/20Y for <10 Y reads in females (9.2% of sex-linked SNPs), 100X/100Y for <20 Y reads in females (0.3% of sex-linked SNPs) and 200X/200Y for <30 Y read in females (0.008% of sex-linked SNPs) using empirical information from known XY genes analysis. Cases where homozygous females had more than 30 reads with the Y allele were excluded.
